# Tumor Microenvironment-Responsive Drug Delivery Based on Polymeric Micelles for Precision Cancer Therapy: Strategies and Prospects

**DOI:** 10.3390/biomedicines12020417

**Published:** 2024-02-11

**Authors:** Zhu Jin, Majdi Al Amili, Shengrong Guo

**Affiliations:** Shanghai Frontiers Science Center of Drug Target Identification and Delivery, School of Pharmaceutical Sciences, Shanghai Jiao Tong University, Shanghai 200240, China; majdi92@sjtu.edu.cn

**Keywords:** tumor microenvironment, cancer therapy, stimuli-responsiveness, drug delivery, precision

## Abstract

In clinical practice, drug therapy for cancer is still limited by its inefficiency and high toxicity. For precision therapy, various drug delivery systems, including polymeric micelles self-assembled from amphiphilic polymeric materials, have been developed to achieve tumor-targeting drug delivery. Considering the characteristics of the pathophysiological environment at the drug target site, the design, synthesis, or modification of environmentally responsive polymeric materials has become a crucial strategy for drug-targeted delivery. In comparison to the normal physiological environment, tumors possess a unique microenvironment, characterized by a low pH, high reactive oxygen species concentration, hypoxia, and distinct enzyme systems, providing various stimuli for the environmentally responsive design of polymeric micelles. Polymeric micelles with tumor microenvironment (TME)-responsive characteristics have shown significant improvement in precision therapy for cancer treatment. This review mainly outlines the most promising strategies available for exploiting the tumor microenvironment to construct internal stimulus-responsive drug delivery micelles that target tumors and achieve enhanced antitumor efficacy. In addition, the prospects of TME-responsive polymeric micelles for gene therapy and immunotherapy, the most popular current cancer treatments, are also discussed. TME-responsive drug delivery via polymeric micelles will be an efficient and robust approach for developing clinical cancer therapies in the future.

## 1. Introduction

Cancer encompasses a range of conditions marked by the unregulated proliferation of abnormal cells with the potential to infiltrate surrounding tissues. Globally, it is the primary contributor to mortality, accounting for approximately 7.6 million deaths in 2008, equivalent to nearly 13% of the total fatalities. More recent statistics from 2020 indicate an alarming increase, with approximately 10 million deaths attributed to cancer. Current projections suggest a potential surge in cancer cases, reaching an unprecedented 22.2 million by 2030 [1].

Surgery is the primary treatment modality for cancer, but it is effective only for eradicating tumors in the early stages of the disease [2]. In most cases, patients are required to undergo drug therapy. The therapeutic agents used in cancer treatment include small-molecule chemotherapeutics, peptides, monoclonal antibodies, and others, which need to be delivered to the target site through specific mechanisms to exert their therapeutic effects. However, practical applications often face challenges such as low solubility, short half-life, and instability of these drugs in vivo. Moreover, a lack of target selectivity affects both cancerous and normal cells, leading to severe side effects on tissues such as bone marrow and the gastrointestinal tract. The resulting challenges, including multidrug resistance and a narrow therapeutic index, limit its efficacy. Dose reductions due to side effects further compromise therapeutic outcomes and may contribute to potential metastasis [3,4].

Nanotechnology advancements paved the way for cancer treatment via nanodrug delivery systems [5]. For instance, mesoporous silica nanoparticles [6], gold nanorods [7], liposomes [8], and micelles [9] are extensively utilized for drug delivery and cancer treatment. Among these nanoparticles, micelles, which are made from the self-assembly of amphiphilic polymers, not only increase the solubility of hydrophobic drugs but also increase their biocompatibility, pharmacokinetics, and cellular uptake; extend in vivo circulation; prevent drugs from being quickly decomposed; and protect against enzymatic degradation [10]. Micelles have an outstanding small particle size due to the number of monomers forming a micelle, which is controlled in a thermodynamically dependent manner and formed within a narrow space. The particle size of micelles is crucial since it can impact biodistribution [11,12]. The micellar particle size can be controlled by tuning the structure of the amphiphiles, the aggregation number of the amphiphiles, the molecular weight of the amphiphiles, the synthesis process, and the hydrophilic/hydrophobic segment ratio. The structure of amphiphiles endows micelles with specific properties essential for drug delivery, such as the ability to solubilize hydrophobic drugs in the hydrophobic core, self-assembly, and drug encapsulation by simple physical mixing. The corona of micelles can be tailored to actively target drug molecules at the site of interest by conjugating ligands specific to target tissues or cells, facilitating molecular recognition and interaction between micelles and target cells. Interestingly, altering amphiphilic copolymer components can easily modify several physiological properties, including surface charge, surface properties, and particle size. Other essential properties, such as biodegradation, biocompatibility, and elimination, can also be utilized in amphiphiles [13,14,15].

Solid tumors are characterized by poorly developed blood vessels and hypervascularization containing gaps in the endothelial lining. In addition, the hyperpermeability of the tumor microvasculature allows polymeric micelles to passively diffuse and reach tumor tissues through the secretion of materials and factors from tumors, such as primary fibroblast growth factor, nitric oxide, vascular permeability factors, prostaglandins, vascular endothelial growth factor, and bradykinin [16]. To maximize their tumor-targeting capacity, smart micelles have been developed to control drug release; these micelles remain stable under physiological conditions and in healthy tissue while releasing the drug upon exposure to certain conditions in the cancer niche and unavailable in normal tissue [17]. Solid tumors also exhibit a unique microenvironment, including an acidic pH, a reducing environment, the overexpression of certain enzymes, hypoxia, reactive oxygen species (ROS), and increased adenosine-5′-triphosphate (ATP) levels, which can be harnessed for designing smart stimulus-responsive micelles.

In addition to traditional chemotherapy, gene therapy and immunotherapy have garnered widespread attention for their application in cancer treatment in recent years. The efficient delivery of nucleic acid drugs such as DNA and RNA and immunomodulators, including antibodies, peptides, and small molecules, has become a focal point of research. In synthesizing polymer micelles, the hydrophobic core and hydrophilic shell structure of the micelles can be tailored by selecting appropriate materials, holding significant promise for enhancing both in vitro and in vivo gene delivery efficacy. Cationic polymer-based micelles, specifically those composed of polyethyleneimine (PEI), are being explored as advantageous carriers for gene encapsulation. These micelles, which feature stimulus responsiveness and active targeting, enhance the stability and permeability of genes through cell membranes and specificity. Notably, polymeric micelles exhibit promise in cancer immunotherapy by enhancing the delivery of immunostimulatory agents and improving the pharmacokinetics and biodistribution of immune-modulating drugs. The dual potential of polymeric micelles in gene delivery and cancer immunotherapy highlights their significance in advancing cancer treatment strategies [18,19]. This review summarizes various strategies for tumor microenvironment (TME)-responsive drug delivery using micelles, focusing on pH, redox reaction, enzyme, ROS, and hypoxia responsiveness, and outlines the prospects of polymeric micelles in gene therapy and cancer immunotherapy.

## 2. Tumor Microenvironment

Tumor cells induce substantial molecular, cellular, and structural alterations within their host tissues. The evolving TME is intricate and continuously changing. Although the composition of the TME varies among different types of tumors, common features include immune cells, stromal cells, blood vessels, and the extracellular matrix. It is widely recognized that the TME is not a passive bystander but rather an active facilitator of cancer progression. During the early stages of tumor growth, dynamic and reciprocal interactions occur between cancer cells and TME components, supporting cancer cell survival, local invasion, and metastasis [20]. To counteract hypoxic and acidic conditions, the TME orchestrates an angiogenic program to restore oxygen and nutrient supplies while eliminating metabolic waste. A diverse array of adaptive and innate immune cells infiltrates tumors, exhibiting both pro- and antitumorigenic effects [21].

The TME possesses distinctive attributes that can be harnessed for TME-targeted nanoparticles. Notably, the extracellular pH in the TME tends to be more acidic (pH 6.5 to pH 6.9) than the physiological pH of normal tissue (7.2 to 7.5). This acidity arises from the heightened glycolysis rate in cancer cells, which converts glucose into lactic acid to meet their energy demands. This pH variance in tumor cells offers the potential for designing pH-responsive cancer-targeting systems [22]. Another unique feature is hypoxia, where deep-seated tumor cells suffer from oxygen deprivation due to irregular vascular networks within solid tumors. These slowly proliferating cells in hypoxic regions display reduced susceptibility to conventional antiproliferative drugs [23].

Additionally, the TME exhibits altered expression of specific enzymes, often from the protease family, such as matrix metalloproteinases (MMPs), or from the lipase family, such as phospholipase A2. Enzyme–substrate specificity has spurred the development of enzyme-responsive nanomaterials for targeted drug delivery [24]. Furthermore, tumor cells in the TME face heightened oxidative stress, which is attributed to elevated levels of superoxide anion radicals, hydroxyl radicals, and hydrogen peroxide. To combat this, tumor cells increase their redox potential by expressing redox species such as superoxide dismutase and reduced glutathione (GSH). This imbalance in oxidation and reduction potentials within the TME presents an excellent opportunity for designing TME-targeted nanoparticles that recognize elevated levels of ROS compared to those of normal cells due to their aerobic metabolism resulting from oncogenic transformation [25,26,27]. These inherent TME stimuli offer promising prospects for the development of TME-responsive nanoparticles. While targeting the TME for cancer treatment holds significant promise, current FDA-approved treatments have limited effectiveness. As our understanding of how the TME contributes to tumorigenesis continues to evolve, new therapeutic targets and strategies will undoubtedly emerge.

## 3. Strategies for TME-Responsive Drug Delivery

**Figure 1:**
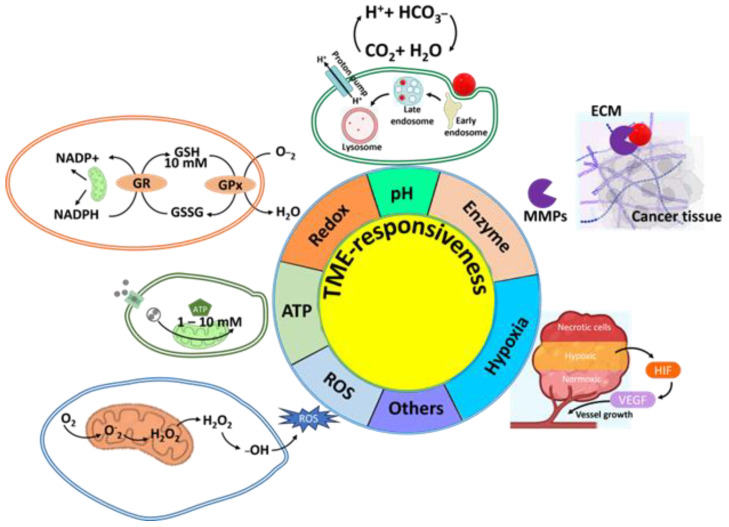
Schematic illustration of TME-responsiveness for drug delivery based on polymeric micelles.

**Figure 2:**
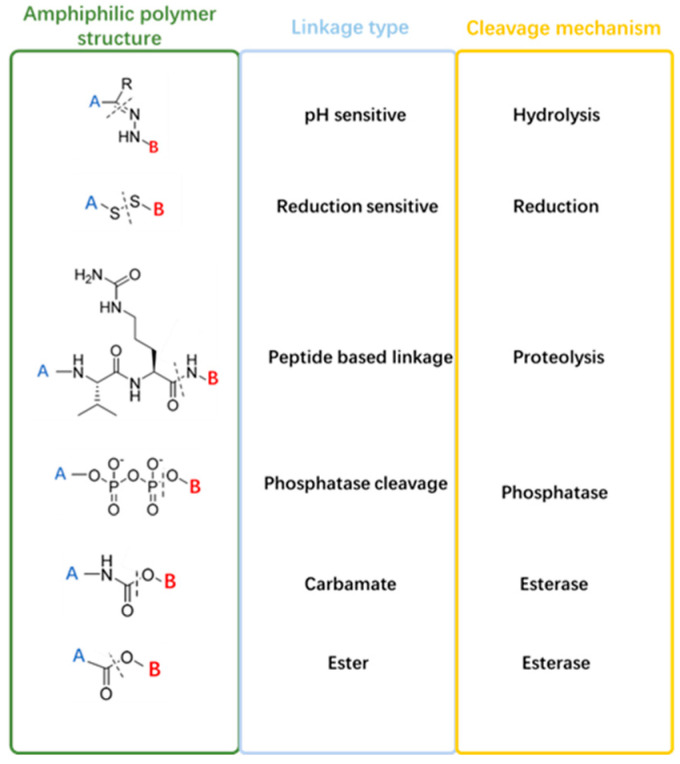
Schematic illustrating amphiphilic polymer structures, linkage types, and cleavage mechanisms of some typical TME-responsive linkers. A and B stand for hydrophilic and hydrophobic segments, respectively.

The amphiphiles of micelles can be specifically designed to respond to internal stimuli such as pH, redox conditions, enzymes, and temperature for tumor-specific drug delivery. The nanoparticles should interact with the tumor site and prevent interaction with healthy tissues, which ideal targeted nanoparticles can offer. Stimulus-responsive micelles are highly preferred for this approach due to their desirable features. They can interact well with the site of action cells and respond to the surrounding tumor microenvironments, such as through changes in pH, enzymes, or redox [28]. The mainstream strategies for TME-responsive drug delivery are summarized as shown in Figure 1. Some typical stimulus-sensitive cleavage linkers that can be exploited to construct TME-responsive amphiphilic polymers are listed in Figure 2 [29].

### 3.1. pH Responsiveness

**Figure 3:**
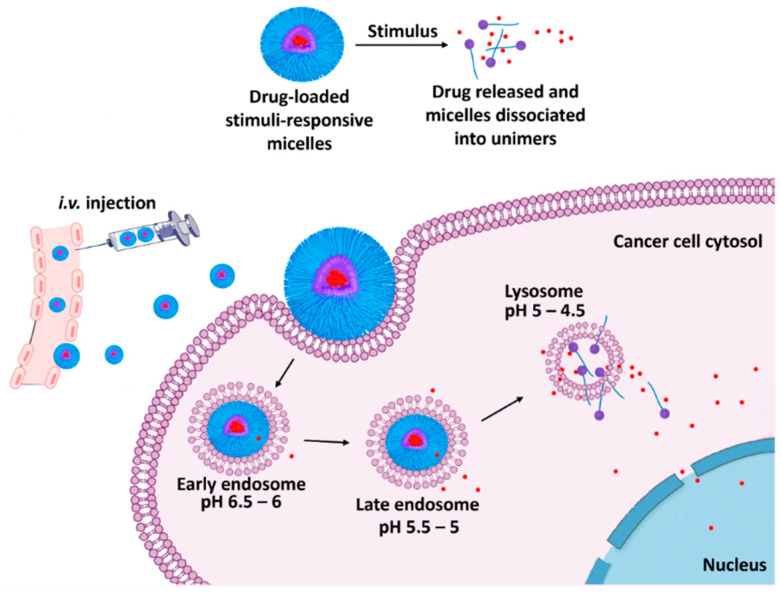
Schematic illustration showing drug release via micelle dissociation under different internal acidic conditions. Red dots are the released drug. Following micelles’ endocytosis, they are entrapped within the early endosome, late endosome, and then the lysosome. Red dots are the released drug, concentrated in the lysosomal area due to the highest acidity level.

The human body sustains a steady pH of 7.4 in healthy physiological tissues [30]. Moreover, tumor disease sites have a pH of 5~6 due to the accumulation of lactic acid that results from the rapid division of cancer cells [31]. This pH variation can be exploited to release the cargo of micelles in a controlled manner exclusively at the tumor site in response to its acidic pH (Figure 3) [32]. Cargo release can be accomplished by either breaking the labile bond, which forms the amphiphile of micelles, or destabilizing micelles via alteration of the size, shape, or hydrophilic–hydrophobic balance. Diverse pH-responsive micelles have been investigated and developed for cancer treatment. The strategy for selecting polymers relies on the existence of ionizable chemical groups. These polymers can be classified into anionic pH-responsive micelles and cationic pH-responsive micelles depending on the content of the ionizable chemical group of polycarboxylic or polyamine groups. To select a suitable polymer that is usually a weak acid or base, it should be considered whether its pKa is suitable for the desired pH at the site of action [33]. A negatively charged polymer containing carboxylic groups is used to design anionic pH-responsive micelles. The carboxylic acid groups are protonated (nonionized) at basic pH with hydrophobic properties. They can be deprotonated under acidic conditions (ionic form) while being hydrophilic at acidic pH. This change from hydrophobic to hydrophilic allows destabilization of the micellar system. This results in cargo release at tumor sites. Polymers with these characteristics usually contain carboxylic acid groups such as polymethacrylic acid, poly(acrylic acid) (PAAc), polyglycolic acid, and polyglutamic acid. In addition, polymers consisting of sulfonic acid groups such as poly(4-styrene sulfonic acid) and poly(2-acrylamido-2-methylpropane sulfonic acid) are also utilized as negatively charged polymers [34].

These polymers are usually desired for the synthesis of hydrogels capable of shrinking and swelling in a pH-dependent manner to achieve cargo release. A positively charged polymer, which contains polybases such as PEI and polyamines, is used to synthesize these types of micelles. The amine group in the polymeric chain can accept protons at acidic pH and donate protons at basic pH. Cationic pH-responsive micelles can improve cellular uptake due to their positive surface charge resulting from ionizable polyamines, for instance, poly(*N*,*N*′-dimethylamino ethyl methacrylate) and PEI. Tuning the pKa of the cationic amine groups allows the polymers to be protonated at acidic pH and deprotonated at basic pH. This charge alteration destabilizes the micellar complex and triggers cargo release. Unfortunately, cationic polymers are considered more cytotoxic than anionic polymers due to their ability to interact with negatively charged proteins during blood circulation non-specifically. Moreover, positively charged micelles cause serum inhibition, rapid clearance, and instability with opsonin. Anionic polymers are impeded for efficient cellular uptake due to cellular repulsion by negatively charged plasmalemma. Charge-reversal pH-responsive micelles, which can be transformed from negatively to positively charged micelles and vice versa in a pH-dependent manner, have recently been developed. This approach’s advantage is achieving active tumor targeting without a specific targeting ligand [35].

**Figure 4:**
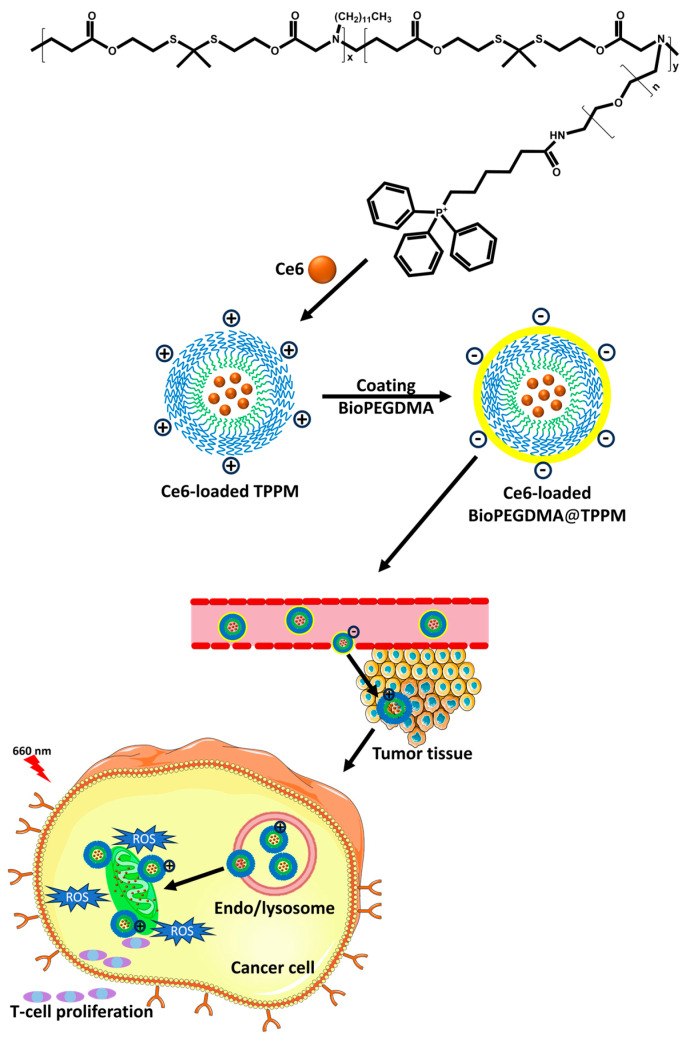
Schematic illustration showing a pH-responsive micelle of BioPEGDMA@TPPM micelles for enhanced PDT. The self-assembly and Ce6 loading were followed by coating with BioPEGDMA via electrostatic interaction, resulting in tumor-targeted delivery and charge reversal in TME. The endo/lysosomal escape, mitochondria targeting, generation of ROS under laser irradiation, and stimulated immune responses based on BioPEGDMA@TPPM.

Moreover, it increases the circulation half-life of micelles in the blood, enhances cellular uptake, and accomplishes efficient drug release in target cells [36]. Peng et al. designed charge-reversal micelles consisting of positively charged micelles composed of an amphiphilic copolymer core with a mitochondrial active targeting moiety (triphenylphosphonium) (TPP) and denoted as Ce6@TPPM. Furthermore, the pH-responsive outer layer consisted of anionic 2,3-dimethyl maleic anhydride (DMA)-conjugated biotin-polyethylene glycol (PEG)-NH_2_ for active tumor targeting and Ce6 delivery, denoted as Ce6@TPPM-BioPEG-DMA. The system was stable at pH 7.4 in physiological environments with a negatively charged surface (Ce6@TPPM-BioPEG-DMA). Upon reaching the acidic tumor microenvironment, the system efficiently accumulated due to ligand–receptor-mediated active targeting; subsequently, the system converted to a positively charged layer (Ce6@TPPM) at pH~6.5, which further accelerated the accumulation of micelles in the tumor tissue and allowed the TPP to be re-exposed inside tumor cells to actively target the mitochondria (Figure 4) [37]. This strategy overcomes the cytotoxicity and rapid clearance of positively charged micelles and enhances the cellular uptake of negatively charged micelles.

### 3.2. Redox Responsiveness

The difference in the concentrations of the tumoral reductants, which are represented mainly by GSH, was approximately 2–10 × 10^−3^ M, especially in multidrug-resistant tumors. Moreover, the GSH concentration in the extracellular fluid ranged from 2 to 10 × 10^−6^ M. There are three prominent redox couples, GSH/GSSG [38], NAD(P)H/NAD(P)^+^ [39], and Tex(SH)_2_/TrxSS [40], which exist in the TME. Redox-responsive micelles containing redox-sensitive moieties have been designed to deliver and release cargoes exclusively at tumor sites in response to tumor-reductive environments.

The most commonly employed redox-responsive polymer is a disulfide bond-containing polymer that can rapidly respond to and be cleaved by redox components such as GSH. Disulfide bonds can be readily cleaved by GSH to form a sulfhydryl group, leading to destabilization of the micellar system and cargo release. Rapid cleavage of disulfide bonds (within minutes to hours) is more favorable than long cleavage periods (from weeks to months), represented by polycarbonates and aliphatic polyesters, due to rapid intracellular drug release, which is advantageous for inhibiting cancer cell growth during the first hours after injection [41]. Diselenide (Se-Se) and carbon–selenide (C-Se) bonds have also attracted attention from many studies due to their lower bond energies (Se-Se 172 kJ/mol and C-Se 244 kJ/mol) [42,43], which require less energy for bond cleavage than disulfide bonds (S-S 268 kJ/mol) [44]. Moreover, the maleimide–thioester bond (C-S 272 kJ/mol) exhibited increased blood stability and decreased cargo release [43].

**Figure 5:**
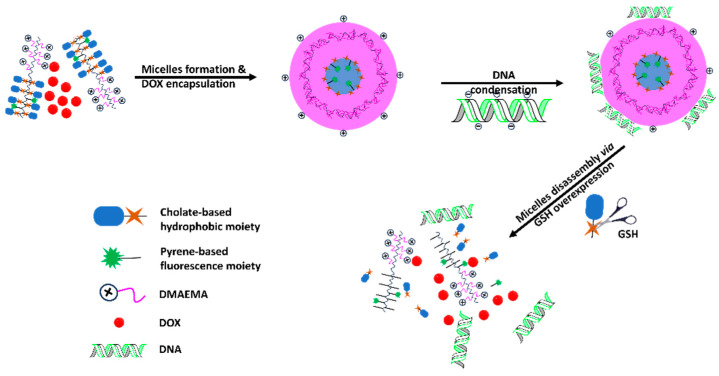
Schematic illustration showing the micelles’ self-assembly and DOX loading followed by DNA condensation. DOX and DNA are released upon GSH overexpression in the TME.

Sahoo et al. fabricated the most promising redox-responsive micellar system by self-assembly of poly(*N*,*N*′-dimethylaminoethylmethacrylate)-*b*-(poly(2-(methacryloyl)-oxyethyl-2′-hydroxyethyl disulfidecholate)-*r*-2-(methacryloyloxy)ethyl-1-pyrenebutyrate). The anticancer agent doxorubicin (DOX) was encapsulated in the micellar core, and DNA was complexed with the outer layer of micelles to form micelleplexes. In the absence of GSH, 8~10% of the DOX was released within 48 h. Moreover, in the presence of 10 mM GSH, 90% of the DOX was released, confirming the cleavage of disulfide bonds that conjugate the hydrophobic cholate group with the polymeric backbone, allowing DOX molecules to be released into the tumor medium due to the disassembly of micelles (Figure 5) [45]. An earlier study in 2018 showed the least drug leakage in a formulation based on redox-responsive drug release [46]. The authors achieved less than 5% leakage of paclitaxel (PTX) within 48 h by conjugating the drug to the hydrophobic part of the amphiphilic block copolymer to form PEG-*b*-poly(5-methyl-5-propargyl-1,3-dioxan-2-one)-*g*-PTX, which self-assembled into micelles for the treatment of HeLa tumor-bearing mice. However, in the presence of 10 mM dithiothreitol (DTT), the release reached 70%. Briefly, the advantage of redox responsiveness is stability in healthy tissues, which results in fewer side effects and cytotoxicity.

Disulfide crosslinking chemistry was extensively used for not only small-molecule drugs but also macromolecular therapeutics such as nucleic acids. It was extensively utilized in advancing the development of polyplex-based carriers to deliver a wide range of cargo molecules, such as plasmid DNA, siRNA, and mRNA. Interestingly, the introduction of charge-preserved disulfide crosslinking via 1-amidine-3-mercaptopropyl groups presented high protection to packaged nucleic acids against enzymatic degradation compared to charge-compensated crosslinking via 3-mercaptopropionyl groups to polycation segment of PEG, thereby facilitating maximized intracellular delivery of nucleic acids [47,48].

### 3.3. Enzyme Responsiveness

Enzymes play essential roles in metabolic and biological processes in the human body due to their catalytic properties and high specificity [49]. In tumors, several enzymes are dysregulated and overexpressed in cancer cells. Exploiting these overexpressed enzymes as triggers for cargoes loaded in micelles can be achieved by incorporating enzyme-responsive moieties into the side chain or main chain of the micelles that can be degraded by these enzymes in either the intracellular or extracellular tumor microenvironments to release the cargo [50,51]. There are two main types of enzyme-responsive micelles: oxidoreductases and hydrolyzed micelles.

Oxidoreductases, such as oxygenases (oxygen transfer from molecular oxygen), oxidases (electron transfer to molecular oxygen), peroxidases (electron transfer to peroxidases), and dehydrogenases (hydride transfer), function as catalysts for oxidation–reduction reactions. Oxidoreductases have been exploited for enzyme-responsive drug release due to the oxidative environments they can produce in many diseases, including cancer. The ability of oxidoreductases to catalyze the transfer of electrons between biological molecules requires the presence of an enzyme cofactor, which can function as an electron carrier, such as NAD^+^ or NADP^+^; accordingly, the electron donor is a reductant. In contrast, the electron acceptor substrate is an oxidant [52].

**Figure 6:**
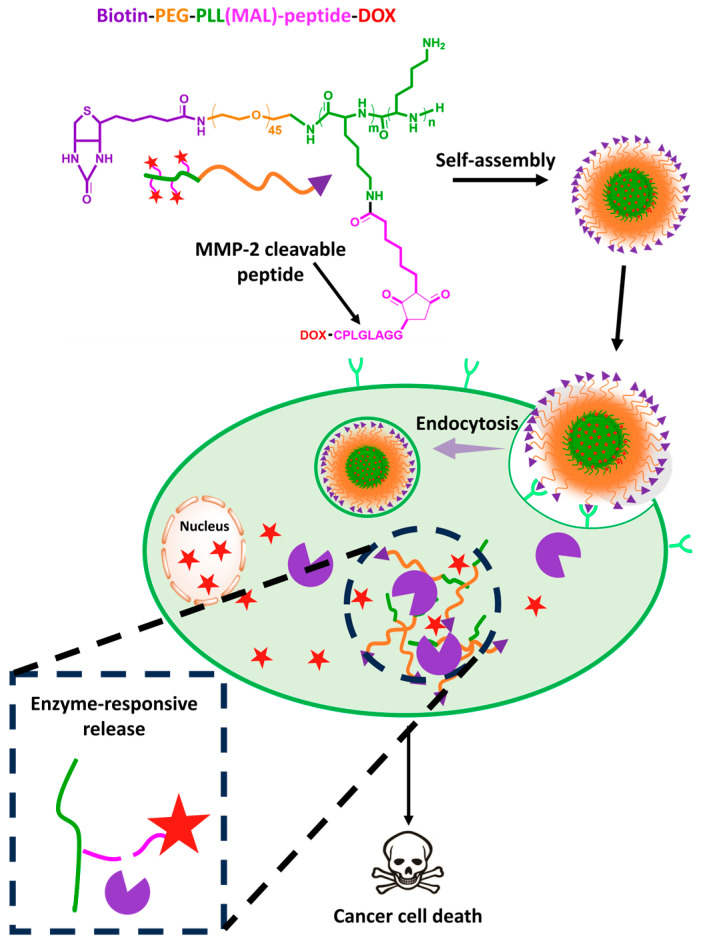
Schematic illustration of the formation of MMP-2-responsive polymeric micelle-based biotin-PEG-*b*-PLL(Mal)-peptide-DOX amphiphilic and intracellular delivery. The red star stands for DOX.

On the other hand, some hydrolysis enzymes are also overexpressed in many stages of human cancers and are involved in cancer initiation, progression, angiogenesis regulation, and metastasis [53]. Generally, MMPs are the most utilized enzymes for stimuli-responsive drug delivery. Chen et al. developed a promising micellar nanoplatform formed by the self-assembly of the biotin-PEG-block-poly(*L*-lysine)(Mal)-peptide-DOX (biotin-PEG-*b*-PLL(Mal)-peptide-DOX) amphiphilic copolymer for the treatment of mouse squamous cell carcinoma and African green monkey kidney fibroblast cells (Figure 6). The peptide is an MMP-2-sensitive linker that can be cleaved in the presence of the MMP-2 enzyme to release DOX in the tumor milieu. The presence of the MMP-2 enzyme induced 46.2% DOX release within 6 h, and almost no drug leakage occurred in the absence of MMP-2 or MMP-2 in combination with the inhibitor [54]. This system has precise enzyme responsiveness and active targeting properties via biotin ligands and can be considered among the most promising nanoplatforms for safe and precise drug delivery. Despite newer studies reporting that 30% [55], 40% [56], and 62.5% [57] of the loaded drugs leaked out in the absence of MMP-2, these drugs cannot be considered to have precise responsiveness or safety for clinical use compared with the biotin-PEG-*b*-PLL(Mal)-peptide-DOX formulation. Cathepsin B is one of the most widely overexpressed cysteine cathepsins in various cancers, and it is involved in the degradation of fibronectin, type IV collagen, and laminin, which leads to cell migration, invasion, and angiogenesis [58].

A micellar nanoplatform was formed by the self-assembly of the [(DEAMEMA)-c-(BMA)]-*b*-[(PEGMA_300_)-c-(peptide)] amphiphile, which is responsive to cathepsin B. The BIM peptide was conjugated to the amphiphile through the FKFL peptide linker, which can be cleaved in the presence of cathepsin B to release BIM specifically in the endolysosome/lysosomes of tumor cells. To confirm the cathepsin B-triggered cleavage of FKFL, the authors performed a cathepsin B cleavage assay; then, the cleavage was quantified via RP-HPLC and mass spectrometry. As a result, cathepsin B rapidly and specifically cleaved the FKFL linker to release BIM in the endosome of SKOV3 ovarian cancer cells and induced apoptosis to kill cancer cells [59]. Phospholipase A2 (PLA2) is overexpressed in several types of tumors at 22-fold higher concentrations than those in normal tissues, especially in prostate cancer [60]. Gao et al. designed PLA_2_-responsive phospholipid micelles loaded with superparamagnetic iron oxide nanoparticles (SPIONs) to successfully release drugs in response to PLA_2_ and via noninvasive magnetic resonance imaging (MRI) [61]. A later study reported biocompatible upconversion nanoparticle (UCNP)-loaded phosphate micelles for bioimaging prostate cancer cells. The release of UCNPs via PLA-2-responsive cleavage was achieved exclusively at tumor sites rather than in healthy cells [62].

### 3.4. ROS Responsiveness

ROS, such as hydrogen peroxide (H_2_O_2_), singlet oxygen, superoxides, hypochlorite ions, peroxynitrites, superoxide anions, and hydroxyl radicals, are byproducts produced by electron transport reactions in the mitochondria of healthy cells [63,64]. They are important for metabolism and intercellular signal functioning and exist at low concentrations of approximately 20 × 10^−9^ M. In cancer cells, these levels increase 1000-fold due to abnormal metabolism, mitochondrial malfunction, and oncogene expression, which result in abnormal metabolism, proliferation, and survival [65]. This difference can be exploited to trigger the release of drug-loaded micelles in the tumor microenvironment specifically. ROS-responsive micelles can be developed by employing various ROS-sensitive materials, such as thioketals, thioethers, ferrocene groups, boronic esters, and sulfides [66]. These materials can undergo certain reactions, such as hydrophobic-to-hydrophilic or hydrophilic-to-hydrophobic transitions, upon elevated ROS levels in tumor microenvironments, leading to micellar system destabilization and drug release. Thioketal is widely used as a ROS-sensitive linker due to the ease of cleaving this linkage [67]. For example, Wang et al. developed micelles for prostate-specific membrane antigen-negative (PISMA (-)) prostate cancer treatment. The micellar system was made by the self-assembly of two copolymers. First, the DUP-1 peptide was conjugated with PEG-1,2-distearoyl-sn-glycero-3-phosphoethanolamine (DUP-PEG-DSPE). Second, DOX and TK linkers were decorated with the side chain of PEG-*b*-PLL to produce a ROS-responsive polymer prodrug (P(L-TK-DOX)) to form a micellar system loaded with a ROS generation agent (α-tocopherol succinate, α-TOS). The micellar system was shown to accumulate and internalize cancer cells via the DUP-1-targeting peptide specific for PSMA (-). α-TOS release upon exposure to elevated concentrations of ROS in the tumor microenvironment could further elevate ROS levels to induce toxicity and enhance ROS responsiveness. Interestingly, less than 5% of the DOX leaked out in the absence of ROS. Moreover, in the presence of different ROS concentrations (20 nM, 0.1 mM, and 1 mM H_2_O_2_), the DOX release increased to 18%, 57%, and 79%, respectively, within 48 h. The study conclusion revealed that the combination of ROS-sensitive drug release behavior and active targeting of tumors is an effective treatment for human PSMA(-) prostate cancer [68].

### 3.5. Hypoxia Responsiveness

Hypoxia is involved in many diseases, such as cardiovascular disorders, rheumatoid arthritis, anemia, and cancer [69]. The oxygen level decreases due to rapid cancer cell growth and insufficient blood supply, which results in tumor angiogenesis and metastasis. An abnormal vascular network is incapable of providing adequate oxygen to cancer cells, especially in the center of the tumor region, which results in an acute hypoxic or oxygen deficiency gradient, which increases from tumor terminals or blood vessels to the tumor center. Hypoxia is a significant stimulus that triggers drug release due to its rarity in normoxic cells [70]. However, the distance between hypoxic region blood vessels and increased efflux transporters prevents the influx of nanoparticles to these hypoxic regions [71]. To overcome this dilemma, several strategies exist for developing drug delivery systems that can provide deep penetration into the hypoxic region. The accumulation of nanoparticles in hypoxic regions could be facilitated by developing nanoparticles upon size-, shape-, and charge-dependent uptake via passive diffusion [72]. Active targeting of nanoparticles is a robust and efficient method to deeply penetrate tumor sites by conjugating targeting ligands such as the cyclic peptide internalizing RGD (iRGD) on the outer surface of the nanosystem or therapeutic drug to increase their ability to penetrate the neuropilin-1 receptor, which is overexpressed in tumor cells and angiogenetic blood vessels [73].

Hypoxia is also involved in the induction of radioresistance, chemoresistance, and cancer recurrence through hypoxia evasion of apoptosis, inactivity of stem cells, and dysregulation of the cell cycle [74]. Hypoxia-bioreductive prodrugs or hypoxia-activated prodrugs, also known as hypoxia-selective cytotoxins (for instance, quinone derivatives such as mitomycin C, N-oxide derivatives such as banoxantrone dihydrochloride and tirapazamine, and nitroimidazole derivatives such as 2-nitroimidazole), are inactive compounds that are spontaneously converted to cytotoxic substances upon specific metabolic pathways that exist in the hypoxic microenvironment [75]. This conversion can be employed directly to kill cancer cells. In addition, upon the prodrug’s conversion from one property to another, a micellar system containing these prodrugs is constructed to trigger drug release, specifically in hypoxic tumor regions, resulting in micelle destabilization and drug release.

### 3.6. Other Stimulus Responsiveness

Cancer cells tend to absorb large amounts of glucose to promote tumor growth, metastasis, and survival [76,77]. Therefore, glucose-responsive micelles can be designed by incorporating glucose oxidase (GOx) within the polymeric chains of the micellar system. Upon reaching the tumor microenvironment, a competitive combination of GOx and glucose destabilizes the micellar system and triggers drug release in cancerous tissues [78]. Most of the applied strategies involve treating diabetes. However, several cancers are involved in diabetes and glucose metabolism. A glucose-responsive nanosystem can indirectly trigger drug release via the conversion of glucose to gluconic acid by the catalysis of GOx, which decreases the pH of the medium and triggers release. Another strategy for killing cancer cells via glucose-responsive treatment is carried out by competing with cancer cells for glucose consumption and converting glucose to gluconic acid and H_2_O_2_; this approach is known as cancer starvation therapy. H_2_O_2_ is essential for physiological processes, such as cell growth and the immune response. Moreover, increasing concentrations of H_2_O_2_ resulted in increased cytotoxicity and cancer cell death [79].

ATP is present in all organisms, is involved in the production and degradation of many cellular compounds, and is the primary source of cellular energy for signaling and metabolism. A substantial concentration of ATP was observed in the intracellular compartment (∼3 mM) compared to the extracellular environment (∼0.4 mM) [80]. Utilizing this difference in ATP concentration gradient between extracellular and intracellular spaces, ATP-responsive drug delivery systems were engineered, which stably encapsulate the therapeutic cargoes in the extracellular medium, smoothly releasing those cargoes after exposure to the high-ATP concentration milieu of the cytosol [81]. For example, a polyplex micelle was formulated with reversible phenyl-boronate ester linkages with phenylboronic acid moieties in the block copolymers and polyol moieties of oligoRNAs hybridized with mRNA in the polymeric micelle (PM) core. This design substantially protected the mRNA from enzymatic degradation in the extracellular space and efficiently released entrapped mRNA to the cytosol for efficient translation.

Polymers that undergo phase transitions upon exposure to certain salt concentrations exhibit reduced electrostatic strength due to the high salt concentration, making these polymers ionic strength- or salt-responsive materials. An increase in salt concentration decreases the electrostatic repulsion between the copolymers, leading to precipitation and drug release [82]. Salt-responsive materials exhibit unusual rheological behavior due to the attractive Coulombic interactions between oppositely charged species, which cause alterations in the solubility, size, length, and surface charge of the polymer [83,84]. These materials respond to the ionic strength of PAAc and methacrylic acid, which undergo viscosity changes and shrinkage upon exposure to high salt concentrations due to the attraction pairs of the ions [85]. Compared with those in normal lactating breast epithelium, salt concentrations in breast cancer tissues were significantly greater. Brain cancers also exhibit an influx of intracellular sodium ions to promote tumor cell proliferation. The epithelial sodium channel (ENaC) regulates the entry of sodium into the intracellular compartment. Abnormalities in ENaC function correlated with tumors result in antiapoptotic effects, uncontrolled tumor growth, cell migration, and angiogenesis [86]. Exploiting the difference in salt concentration between the tumor microenvironment and healthy tissue to construct ionic strength-responsive drug delivery systems has not been widely investigated.

### 3.7. Multistimulus Responsiveness

**Figure 7:**
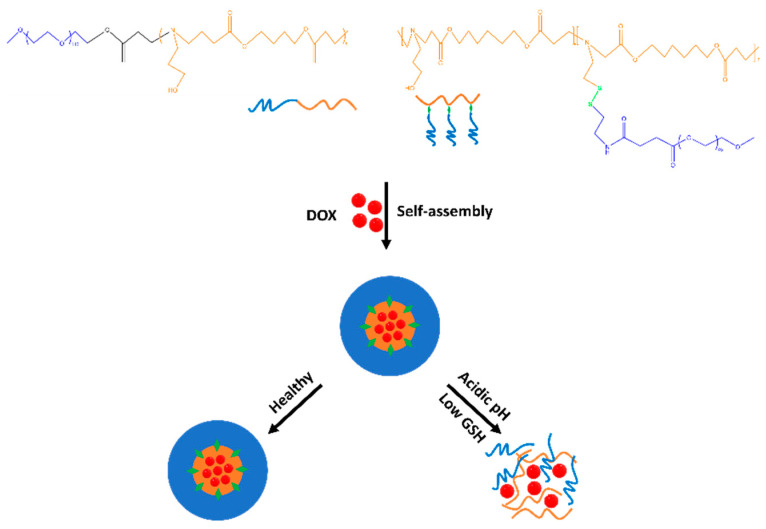
Schematic illustration indicating the co-micellization of pH/redox dual-responsive diblock copolymers for drug delivery and controlled release triggered by acidic pH and overexpression of GSH in TME.

Polymeric micelles that are responsive to multiple stimuli are gaining prominence and demonstrate significant promise for targeted drug delivery and cancer therapy. The integration of various sensitivities into a single polymeric micellar system allows more precise control of drug delivery and release, leading to enhanced anticancer activity both in vitro and in vivo. These combined sensitivities to different stimuli can occur simultaneously or sequentially, offering versatility in therapeutic applications. Luo et al. designed pH- and redox-responsive PMs to deliver the anticancer drug DOX (Figure 7). The present study investigated the pH sensitivity of the PMs by determining the pKb values, which were 6.45, 6.57, and 6.72 for PM-1, PM-2, and PM-3, respectively. The low critical micelle concentration (CMC) values (3.1 mg/L, 4.2 mg/L, and 6.4 mg/L) indicated the thermodynamic stability of the polymeric micelles, which made them efficient drug carriers. The redox responsiveness of the PMs was evaluated through size and zeta potential changes in the presence of DTT, which demonstrated increased particle sizes due to the cleavage of disulfide bonds. The in vitro drug release profiles of DOX-loaded PMs were examined at different pH values and in the presence of DTT. The results indicated that controlled drug release was triggered by specific microenvironmental cues, such as pH and reducing agents. Cytotoxicity assays revealed that blank PMs had negligible cytotoxicity, while DOX-loaded PMs exhibited greater cytotoxicity against HepG2 cells than free DOX [87]. Zhang et al. designed a multifunctional polymeric system for dual-enzyme- and redox-triggered intracellular drug release to improve cancer treatment efficacy. The key components of their system were the enzyme-responsive polymer PBA-PEG-Azo-PCL and the redox-responsive prodrug mPEG-ss-CPT. Azo bonds in PBA-PEG-Azo-PCL were shown to be cleaved by azoreductase and the coenzyme NADPH, mimicking the tumor tissue microenvironment. The micelles exhibited highly sensitive tumor microenvironment responsiveness, with changes in size indicating successful cleavage of the azo bonds. Additionally, the disulfide bonds in mPEG-ss-CPT were cleaved in the presence of GSH, increasing the micelle size. The dual-responsive behaviors were explained by a series of chemical reactions, ensuring controlled drug release. In vitro drug release studies demonstrated that the micelles exhibited good stability in blood circulation but rapidly released their cargo inside tumor cells, particularly under conditions mimicking the tumor microenvironment. In vivo and ex vivo fluorescence imaging confirmed the selective accumulation of the micelles at the tumor site. The dual-responsive micelles exhibited significant anticancer activity with minimal side effects on normal tissues, as demonstrated by tumor volume changes, survival rates, and histological analyses [88].

## 4. Prospects in Cancer Therapy

### 4.1. Gene Therapy

Gene delivery requires appropriate carriers with high gene transfer efficiency, good biocompatibility, and low cytotoxicity. Genetic materials such as plasmid DNA (pDNA), siRNA, and RNA demand cationic polymers to successfully complex these negatively charged genetic agents with cationic polymer-based micelles [89]. Naked genetic materials suffer rapid elimination from the body, instability in the blood circulation, and inability to diffuse through the cell membrane, ascribed to their large anionic charge. Consequently, a nanocarrier is required to entrap these genes with a high buffering capacity for improved transfection and the capability to efficiently target diseased cells and release the genes in the intracellular compartment, allowing the siRNA to target the cytoplasm and DNA to target the nucleus. Employing viral carriers to encapsulate genes is unfavorable due to their potential genotoxicity and chance of producing replication-competent viruses [90]. Cationic polymers have been extensively used to deliver nucleic acids through their electrostatic polyionic self-assembly with nucleic acids, allowing the polyion complex formation, named “polyplex” [91]. Polyplex micelles are stealth-polymer shielded nucleic acid delivery systems constructed through electrostatic self-assembly between nucleic acids and polycationic block copolymers of PEG or poly(oxazoline), where the nucleic acid is packaged with polycations as the core compartment and the stealth polymer chains surrounding the core as the protective shell compartment. This characteristic core–shell architecture of polyplex micelles protects the nucleic acid cargoes from hydrolytic and enzymatic degradation, which increases the stability and permeability of the nucleic acids through the cell membrane. Surface modification of PEG (PEGylation) onto nucleic acid delivery carriers is a well-known strategy for extending blood retention and improving therapeutic outcomes in vivo. However, PEG shells often present a trade-off between prolonged blood retention and promoted transfection because high-stealth shielding is advantageous in prolonging blood circulation, whereas it is disadvantageous in obtaining efficient transfection due to low cellular uptake and inefficient endosomal escape (PEG dilemma) by minimizing the interactions with the plasma membrane before internalization and endolysosomal membranes after internalization of the targeted cells. To overcome this PEG dilemma, Nishiyama et al. developed stepwise acidic pH-responsive plasmid DNA delivery nanocarriers with a surface covered by ethylenediamine-based polycarboxybetaines. These nanocarriers switched their surface charge potential from a neutral charge at pH 7.4 to a positive charge at tumoral pH 6.5 and endolysosomal pH 5.5, thereby promoting cellular uptake and increasing the endosomal escape toward efficient gene transfection [92]. In another strategy, cyclic RGD (Arg-Gly-Asp) (cRGD) peptide as a ligand was introduced to the distal end of the PEG chains to improve the specific integrin-mediated uptake of disulfide core-crosslinked polyplex micelle-based gene carriers [93,94].

Utilizing smart micelles with stimulus responsiveness and active targeting can lead to robust, highly specific, and optimal development of gene delivery strategies. Polycations made of PEI for gene delivery are among the most common polycations because they exhibit high transfection ability, attributed to the high buffering capacity of the polycation due to the proton sponge effect, by which the polycation buffers endosomal acidification and escalates endosomal ion osmotic pressure due to protonation of the amine groups, resulting in rupture of the membrane of the endosome and the subsequent release of the captured system into the cytoplasm. Accordingly, polycations also demand a high N/P ratio (molar ratio of amino groups (N) in polymer to phosphate groups (P) in nucleic acid) to form a secured complex, resulting in high stability and transfection ability [95,96].

A novel dual-responsive PEI-based polymeric micelle system has been developed for the targeted delivery of therapeutic agents in cancer treatment. Overcoming challenges such as mucosal barriers, nonspecific uptake, and intracellular drug resistance is crucial for achieving high therapeutic efficiency. The key feature of this system is the presence of a sheddable PEI shell, which responds to variations in extracellular pH and intracellular GSH levels. The micelle system exhibited ultrasensitive negative-to-positive charge reversal in response to the extracellular pH. When exposed to the acidic environment commonly found at tumor sites, the surface charge of the micelles changes from negative to positive. This transformation enhances electrostatic interactions, significantly improving the uptake of micelles by cancer cells. Upon internalization by cancer cells, the micelles encounter another layer of responsiveness. The disulfide linkages within the system can be cleaved by the presence of GSH in the cytoplasm. Importantly, GSH concentrations are often greater in cancer cells than in normal cells. The cleavage of disulfide linkages triggers the deshielding of the hydrophilic PEI shell, leading to the rapid release of the encapsulated therapeutic agent. The mechanism of this dual-responsive polymer micelle involves several stages. Initially, micelles, with their originally negatively charged surface, exhibit prolonged circulation time in the bloodstream. At the tumor site, they take advantage of the enhanced permeability and retention effect, accumulating at higher concentrations. The ultrasensitive negative-to-positive charge reversal that occurs in response to an acidic pH facilitates efficient internalization by cancer cells through electronic interactions and folate receptor (FR)-mediated endocytosis. Furthermore, the micelles escape from lysosomes, a cellular organelle, via a proton sponge effect. Excess intracellular GSH triggers the cleavage of disulfide linkages, resulting in the deshielding of the PEI shell and rapid release of the therapeutic agent into the nucleus [97].

Gao et al. developed pH/redox dual-responsive polyplexes demonstrating promising characteristics for codelivering siRNA and DOX. The polyplex exhibited efficient encapsulation of DOX and siRNA, along with pH-/redox-triggered payload release, facilitated by protonation of PHis and disulfide bond cleavage. Specifically, at an N/P ratio of 7, the polyplex displayed superior payload delivery efficiency, MDR1 gene silencing, cytotoxicity against MCF-7/ADR cells, and more potent inhibition of MCF-7/ADR tumor growth than at higher N/P ratios. This enhanced performance at N/P 7 was attributed to the increased electrostatic attraction between the siRNA and oligoethylenimines (OEIs), which suppressed the release of MDR1 siRNA and OEIs. A stronger electrostatic interaction was crucial for overcoming payload endolysosomal sequestration by OEI-induced membrane permeabilization [98]. Pan et al. synthesized dendrimer micelles self-assembled from two copolymers. First, PEG_2k_-1,2-dioleoyl-sn-glycero-3-phosphoethanolamine (PEG_2k_-DOPE)-conjugated generation 4 polyamidoamine dendrimer (G4-PAMAM-D)-incorporated MDR-1 siRNA (siMDR-1) was used. Second, the PEG_5k_-DOPE-conjugated tumor-specific monoclonal antibody 2C5 (mAb 2C5) was used for chemotherapeutic DOX and gene codelivery. The results revealed significant specific binding between cell surface-attached nucleosome tumor cells and the mAb 2C5, which enhanced cellular uptake and increased cytotoxicity in MDR cancer cell lines [99]. An exciting pH-responsive crosslinked polyplex micelle was engineered for mRNA delivery based on cis-aconitic anhydride-modified poly(ethylene glycol)-poly(L-lysine). This polyplex micelle was stable at pH 7.4, whereas it released the packaged mRNA when the pH was decreased below 6.5 (tumoral pH), thus providing high protein expression in the tumor compared to the commercial transfection reagent PEI [100].

### 4.2. Immunotherapy

Cancer immunotherapy has emerged as a successful treatment strategy following conventional surgical, chemical, and radiotherapeutic approaches [101]. Biological therapy harnesses the body’s immune system to induce an attack on tumor cells, resulting in an antitumor effect. The immune system is trained to identify and target specific cancer cells, enhancing the effectiveness of immune cells in eliminating cancer [102]. Notably, cancer immunotherapy addresses both primary tumor and secondary tumor metastasis by triggering a systemic immune response [103]. Additionally, it can impede tumor recurrence by fostering a cancer-specific memory immune response that becomes activated upon encountering tumor-associated antigens [104]. Among the various immunotherapy modalities explored, immune checkpoint inhibitors (ICIs), chimeric antigen receptor T cells, and oncolytic viruses have been extensively studied for their notable achievements in clinical trials. High-dose interleukin-2 has been among the earliest immunotherapies used to activate T cells. Both ICIs and adoptive cell transfer therapy have been demonstrated to be effective against various malignancies [105,106]. Cancer immunotherapy has been proven to mitigate metastasis, prevent tumor recurrence, and reverse multidrug resistance in tumor cells. Notably, its efficacy has been established for treating head and neck cancer, lung carcinoma, leukemia, breast carcinoma, ovarian carcinoma, renal carcinoma, and bladder tumors [107], as polymeric micelles show promise in the realm of cancer immunotherapy by serving various purposes, such as improving the delivery of immunostimulatory agents and enhancing the pharmacokinetics and biodistribution of immune-modulating drugs [108].

These immunotherapeutics include but are not limited to antibodies, small molecules, peptides, and cytokines. A study developed pH- and enzyme-responsive micelles for PD-1 and PTX codelivery, resulting in synergistic cancer chemoimmunotherapy via antitumor immunity by PTX-induced immunogenic cell death (ICD), while aPD-1 blocks the PD-1/PD-L1 axis to suppress immune escape due to PTX-induced PD-L1 upregulation [109]. Different types of cytokines, such as interleukins, interferons, and colony-stimulating factor (CSF), are employed for immunotherapy. In two studies, pIL-12 was codelivered with DOX via pH-/enzyme-responsive micelles to synergistically enhance NK cells and tumor-infiltrated cytotoxic T lymphocytes to achieve synergistic antitumor immune responses through cancer immunity cycle (CIC) cascade activation and amplification, providing therapeutic antitumor and antimetastatic efficacy [110]; this combination of nanosystems was subsequently used to develop a complete CIC-boosted combinatory strategy for developing immunotherapies against cancer and modulating the polarization of protumor M2 macrophages to activated antitumor M1 macrophages [111]. Interferon cytokine-based Mn and ABZI codelivery activate the STING pathway, mature dendritic cells (DCs), and eventually kill tumor cells via cytotoxic CD8+ T cells and NK cells [112]. Mao et al. loaded M-CSF in pH-responsive micelles. The results showed significant inhibition of tumor growth by promoting T-cell tumor infiltration and reversing the M1/M2 polarization balance within the tumor microenvironment [113]. Li et al. designed polymeric micelles based on the amphiphilic diblock copolymer poly(2-ethyl-2-oxazoline)-poly-(D, L-lactide) (PEOz-PLA) in combination with carboxyl-terminated Pluronic F127, for the codelivery of the antigen ovalbumin (OVA) and the Toll-like receptor-7 agonist CL264 (carboxylated-NPs/OVA/CL264) to lymph node-resident DCs. Surface modification with carboxylic groups endows micelles with endocytic receptor-targeting ability, promoting internalization by DCs through the scavenger receptor-mediated pathway. Adjusting the mass ratio of PEOz-PLA to carboxylated Pluronic F127 in the mixed micelles enabled the release of encapsulated CL264 to the early endosome. This resulted in increased expression of costimulatory molecules and secretion of cytokines stimulated by DCs, contributing to an enhanced immune response. Moreover, incorporating PEOz outside the micellar shell facilitated major histocompatibility complex I antigen presentation by facilitating endosome escape and cytosolic release of antigens. This study validated the efficacy of the system in inducing potent immune responses in vivo. Immunization with this codelivery system in tumor-bearing mice not only significantly inhibited tumor growth but also prolonged survival. The findings highlighted the potential clinical applications of this system as an effective antitumor vaccine for cancer immunotherapy. The study also emphasized the importance of particle surface characteristics in enhancing immune responses and demonstrated the advantages of carboxylated NPs in comparison to other formulations [114].

## 5. Conclusions and Outlooks

This review examines recent advancements in TME-responsive polymeric micelles, which are increasingly utilized for delivering chemotherapeutics and biologics in cancer treatment. We categorize TME-responsive strategies based on the endogenous characteristics of the tumor environment. Currently, genes and immunotherapies are at the forefront of cancer treatment, with TME-responsive micelles contributing significantly to this area and showing potential for further development. Numerous preclinical studies have demonstrated the efficacy of these micelles in targeted drug delivery for precise cancer therapy. However, comprehensive safety assessments, including long-term toxicity, biodistribution, pharmacokinetic, metabolic, and excretion studies, are essential before proceeding to clinical trials.

One challenge in this field is the complexity of the manufacturing and quality control processes required for sophisticated micellar systems, which limits industrial scaling. Several micellar formulations, such as Genexol-PM, have reached clinical trials and received FDA approval for breast cancer treatment. However, achieving the desired therapeutic effectiveness and clinical translation remains challenging. A deeper understanding of micelle–biological component interactions is necessary, particularly concerning biodistribution and microenvironmental responses. Issues such as insufficient stimulus sensitivity and nonspecific distribution can lead to off-target effects. Identifying the most effective stimuli for targeted delivery with minimal off-target effects remains a critical research area.

The development of site-specific, patient-tailored micelles is essential for advancing precision cancer treatment. Designing micelles with targeted therapeutic agents and moieties that align with an individual’s genetic profile can enhance delivery efficiency and ensure treatment safety and efficacy. These micelles, integral to precision medicine, deliver precise therapeutic doses to cancer sites and facilitate disease monitoring through advanced imaging techniques. We anticipate that polymeric micelles will emerge as a robust platform for clinical cancer therapy in the near future.
